# Lateral Wedge Insoles With and Without Contoured Arch Support for People With Knee Osteoarthritis and Foot Pain: A Pilot, Feasibility Randomized Controlled Trial

**DOI:** 10.1002/jfa2.70171

**Published:** 2026-07-13

**Authors:** Michael A. Hunt, Natasha M. Krowchuk, Haocheng Zhang, Michael B. Ryan, Trevor B. Birmingham, Jesse M. Charlton

**Affiliations:** ^1^ Motion Analysis and Biofeedback Laboratory University of British Columbia Vancouver British Columbia Canada; ^2^ Department of Physical Therapy University of British Columbia Vancouver British Columbia Canada; ^3^ Graduate Programs in Rehabilitation Sciences University of British Columbia Vancouver British Columbia Canada; ^4^ Kintec Footlabs Surrey British Columbia Canada; ^5^ School of Mechatronics Systems Engineering Simon Fraser University Burnaby British Columbia Canada; ^6^ School of Physical Therapy University of Western Ontario London Ontario Canada; ^7^ Wolf Orthopedic Biomechanics Laboratory Fowler Kennedy Sport Medicine Clinic London Ontario Canada; ^8^ School of Kinesiology University of British Columbia Vancouver British Columbia Canada

**Keywords:** biomechanics, foot, insoles, knee, osteoarthritis, pain

## Abstract

**Background:**

Lateral wedge insoles (LWIs) are a non‐surgical adjunct treatment for knee osteoarthritis (OA) that has been shown to reduce the knee adduction moment—a biomechanical risk factor for knee OA disease progression. Though worn in one's shoes, little is known of the effects of LWIs use on the foot or ankle. Importantly, it unknown whether their use enhances foot symptoms in people with knee OA and concomitant foot pain, thus questioning the general appropriateness of their use in this subgroup.

**Methods:**

We conducted a pilot, feasibility randomized controlled trial to assess study approaches and general effects of LWIs use in people with knee OA and foot pain. 30 individuals with foot pain and radiographically‐confirmed knee OA were randomized to a 12‐week intervention using either standalone 6‐degree LWIs or LWIs integrated with custom foot and arch support. Feasibility outcomes included: recruitment and retention outcomes; insoles usage, reporting, and comfort; and adverse events. We also examined foot (Foot Function Index pain and difficulty subscales) and knee symptoms (Knee Osteoarthritis Outcomes Severity Score pain and daily function subscales), and gait biomechanics (knee adduction and flexion moment peaks and impulses, ankle/subtalar eversion peaks) before and after the 12‐week intervention.

**Results:**

All feasibility criteria were met, and no major adverse events were reported. Participants found the insoles moderately comfortable (7/10), and most weeks (278/336, 83%) met the acceptable 30 min per day wear time threshold. Though no statistical comparisons were made, changes in clinical and biomechanical outcomes were consistent with previous research; specifically, trends toward improvement in knee pain and function, as well as knee adduction moment magnitudes, in both groups.

**Conclusion:**

Lateral wedge insoles, with or without support, appear safe for people with knee OA and concomitant foot pain. Given positive feasibility and efficacy observations, future research in this area is warranted.

## 1 Introduction

Osteoarthritis (OA) is a prevalent musculoskeletal pathology affecting more than 595 million people worldwide, resulting in billions of dollars in direct and indirect healthcare costs annually, and leading to significant, long‐term reductions in quality of life [1, 2]. Given the lack of a cure, combined with the fact that disease progression is associated with greater personal and economic burdens [3], it is imperative to implement inexpensive treatment approaches that not only ameliorate symptoms, but target OA disease progression.

Large and focused loads applied to the knee joint during dynamic movements such as walking are known to be associated with measures of knee OA disease progression such as cartilage loss measured on radiographs [4] or magnetic resonance images [5], as well as advancement to joint replacement surgery [6]. Specifically, high external knee adduction moment (KAM) magnitudes—a proxy of tibiofemoral joint load distribution—are consistently linked to a greater risk of knee OA disease progression [7], and constitute a common biomechanical target of surgical [8] and non‐surgical interventions [9]. One of the most commonly researched interventions designed to reduce KAM magnitudes is the use of shoe‐worn insoles with a raised lateral edge (termed lateral wedged insoles; LWIs). Meta‐analyses of LWI effects show an average 5%–10% reduction in peak KAM with each step taken [10, 11]. They are also inexpensive, well‐tolerated by users, and evoke minimal to no additional patient time burden. However, biomechanical responses to LWIs are variable, with as many as half showing no change, or even an increase in KAM magnitudes [12, 13], and the magnitude of their effects on pain are dependent on the comparator condition [14, 15].

Greater improvements in pain with LWIs compared to neutral inserts have been shown in a previous clinical trial that screened for biomechanical response during enrollment [16], suggesting that attention to biomechanical response in study design may have both biomechanical and symptomatic benefits. Further, despite LWIs acting directly on the foot‐ankle complex, very little attention to foot assessment has been paid in clinical trials. This oversight is relevant as at least 25% of people with painful knee OA report concurrent foot pain [17], and foot pain in the presence of knee OA adversely affects overall health and increases the risk of knee cartilage degradation [18, 19]. Reporting of changes in foot symptoms with LWIs use is sparse in the existing literature, and we are unaware of any studies that have specifically focused recruitment on people with knee OA and foot pain. It is currently unknown whether the use of LWIs by people with knee OA and concomitant foot pain result in an increase in foot symptoms, leaving questions of overall safety unknown, especially in this subgroup.

Given the potential for LWIs to play an important adjunct role in the clinical management of knee OA, and the methodological limitations of previous research noted above, our primary objectives in the present study were to determine the feasibility of conducting a fully‐powered trial, and to assess the general safety of LWIs use by people with knee OA and foot pain. Our secondary objectives were to assess within‐group changes in clinical and biomechanical outcomes in two types of LWIs—standalone LWIs and LWIs that incorporated individualized contoured arch support throughout the feet—to compare with the known efficacy effects of LWIs in the general knee OA population.

## 2 Materials and Methods

### 2.1 Study Design

This was a two‐arm, parallel‐group pilot feasibility randomized study. Individuals who passed all inclusion and exclusion criteria were invited to participate in a 12‐week intervention consisting of either standalone LWIs, or LWIs integrated with individualized foot support. We aimed to recruit 30 individuals for estimation of pre‐defined feasibility metrics. The study was approved by the institutional Clinical Research Ethics Board, and all individuals provided written informed consent prior to gait screening. The study was registered with clinicaltrials.gov (NCT06251167) prior to any participant recruitment, and study reporting adheres to the Consolidated Standards of Reporting Trials (CONSORT) for randomized pilot and feasibility trials [20].

### 2.2 Participants

Community‐dwelling adults were recruited via five social media advertisements, each 2 weeks in duration. Interested individuals completed an online screening form to assess study appropriateness. Initial inclusion criteria included: (i) at least 50 years of age; (ii) history of knee pain longer than six months, with an average magnitude of at least 3 out of 10 (0 = “no pain”, 10 = “worst pain imaginable”) over the six months prior to recruitment; (iii) self‐reported pain in the same foot/feet as the painful knee(s); and (iv) ability to communicate in English. Initial exclusion criteria included: (i) surgery, intra‐articular injection, or corticosteroid use in either knee in the 6 months prior to recruitment; (ii) presence of a systemic arthritic condition; (iii) any history of knee joint replacement or tibial osteotomy surgery; (iv) any condition (other than knee or foot pain) affecting lower limb function; (v) current use of shoe‐worn insoles, or a plan to acquire insoles or footwear modifications in the next 6 months; and (vi) any previous experience with shoe‐worn insoles that resulted in a self‐ or clinician‐initiated termination of use due to onset of lower limb pain. Participants who passed initial screening were invited to the laboratory for a biomechanical screening.

### 2.3 Biomechanical Screening

Twenty‐two retroreflective markers were positioned on the skin using a modified Helen Hayes marker set [21]. Individuals completed two randomized conditions of walking: with and without 3D‐printed, 6° bilateral sulcus length LWIs. Walking trials were completed in slip‐resistant socks with holes cut to permit view of the toe, ankle, and heel markers. For LWIs trials, double‐sided tape was used to affix the insoles to the underside of the feet before donning the socks. First condition trials were conducted at a self‐selected speed, and trials in the second randomized condition were constrained to the average speed from the first condition ± 5%, as measured by photoelectric timing gates. Marker trajectories were collected at 120 Hz using a 14‐camera motion capture system (Motion Analysis Corp., Rohnert Park, CA) synchronized to two floor‐mounted force platforms (OR6‐6, AMTI, Watertown, MA) sampling at 1200 Hz.

Five trials with clean force platform strikes from each condition that met the walking speed constraints were analyzed using commercially‐available software (Orthotrak, Motion Analysis Corp., Rohnert Park, CA) in order to calculate the KAM impulse (area under the KAM curve). Condition averages were calculated across all five trials, and biomechanical response was calculated as the change in KAM impulse in the LWIs condition compared to the no LWIs condition.

At the conclusion of biomechanical testing, the individual was placed prone on a plinth and images of the plantar surface of each foot were acquired using a tablet device with a structured infrared light scanner (TrueDepth scanner, iPad 11 Pro, Apple Inc., Cupertino, CA), to be used later for manufacturing the intervention insoles.

### 2.4 Radiographic Screening

Individuals underwent bilateral standing, semi‐flexed, postero‐anterior radiograph acquisition. Images were assessed by two trained assessors (NMK and MAH) to categorize knee OA severity according to the Kellgren and Lawrence (KL) grading scale [22].

### 2.5 Study Enrollment and Randomization

Limbs considered eligible for study inclusion were those which: (i) all initial knee and foot inclusion and exclusion criteria were met, (ii) exhibited a minimum 4% reduction in the KAM impulse at the biomechanical screening, and (iii) exhibited a minimum KL grade of 2 on knee radiographic assessment, with greater involvement in the medial than lateral tibiofemoral compartment. In instances where both limbs were eligible, the limb with the knee that experienced the largest KAM impulse reduction with LWIs was selected as the study limb. All subsequent biomechanical and patient‐reported data were limited to the study limb.

The randomization schedule was created by a researcher not involved in assessment (MAH), using equal allocations in random blocks of four or six, and stored on a computer accessible only to them. Once all eligibility criteria were met, group allocations were provided to the research coordinator (NMK) upon request one at a time. The research coordinator provided the insoles manufacturer (Kintec Footlabs, Surrey, BC) with the allocation information, shoe size, usual shoe make and model (to tailor fit), as well as the deidentified code containing the foot scan images. Participants were informed upon enrollment that they would receive one of two types of individualized LWIs, but no information on the design specifics was provided.

### 2.6 Interventions

**FIGURE 1:**
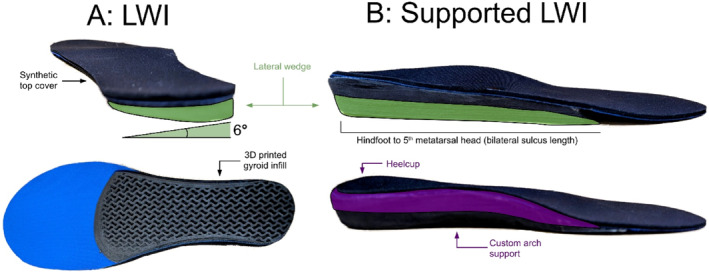
Visualization of insole types. The LWI alone condition (A) was a sulcus‐length (just distal to head of fifth metatarsal) 6° lateral wedge (green). The supported LWI condition (B) used the same sulcus‐length 6° lateral wedge incorporated with contoured arch support (also sulcus length) and heel cupping (purple). Both insoles were manufactured from the same 3D‐printed gyroid infill and finished with the same synthetic top cover. Green and purple outlines are for visualization purposes only—all gyroid infill was the same black colourization for the insoles.

Both insole designs were based on the inclusion of a raised lateral border with a 6° angulation from the posterior aspect to the sulcus (Figure 1). Foot dimensions from the scans obtained during biomechanical screening were used to ensure the insoles matched the appropriate length and width of the feet, and that any plantar contouring was scaled appropriately. All insoles were 3D‐printed with approximately 70% gyroid infill and finished with the same synthetic covering for comfort. The LWI alone condition only included the lateral wedging. The LWIs with support condition also incorporated additional 3D‐printed material to custom arch contour to the plantar aspects of the feet based on data from the 3D structured infrared scans, as well as heel cupping.

### 2.7 Data Collection

Participants were assessed in the laboratory prior to LWIs intervention (Baseline) and after 12 weeks of insoles wear (Week 12), which included completion of patient reported outcome measures as well as biomechanical analysis with and without the allocated insoles.

**FIGURE 2:**
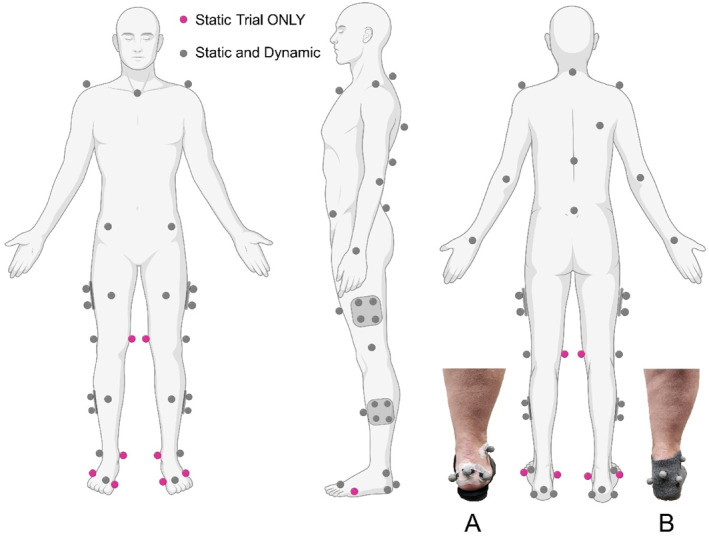
Marker set used for Baseline and Week 12 biomechanics data collection. 53 retro‐reflective markers were positioned over key anatomical landmarks, with 8 of those used only for the initial static standing trial. Insets depict the positioning of the insoles under the foot (A), and enclosure within the socks (B).

Fifty‐three retroreflective markers were affixed to the skin over various anatomical landmarks on the upper and lower body (Figure 2), including eight (bilateral lateral aspects of the first and fifth metatarsals, and bilateral medial epicondyles and malleoli) that were removed after the acquisition of a standing, static trial. Similar methods were used as employed during the biomechanical screening, including: attachment of the insoles to the underside of the feet with double‐sided tape during insoles trials, use of non‐slip socks, and data sampling rates. Self‐selected walking speed was calculated from the first randomly‐selected condition at Baseline and matched for the second condition at Baseline and both at Week 12. In contrast to screening, biomechanical data from Baseline and Week 12 were processed in Visual 3D (HAS‐Motion, Kingston, ON) (see Supporting Information S1 for a description of the biomechanical model).

Upon completion of Baseline testing, and after any insole modifications to enhance fit (e.g., trimming of distal aspect of insoles to properly fit within shoes), the insoles were inserted into the participants' usual shoes, and participants were instructed on proper insoles use and wear. They were provided with access to an online diary to record the following information on a weekly basis: daily insoles wear time (in minutes), insoles comfort (using an 11‐point numerical rating scale with 0 = “completely uncomfortable” and 10 = “completely comfortable”), adverse events, and any qualitative comments related to the intervention. Participants were contacted via email every 3–4 weeks to check in on progress and to discuss any issues identified in the online diary entries.

### 2.8 Outcome Measures

#### 2.8.1 Feasibility Outcomes

The following outcomes were used to assess feasibility, with acceptable criteria for each in brackets: (i) recruitment rate (≥ 5 enrolled participants per recruitment effort); (ii) insoles delivery time (≤ 28 days from request to delivery); (iii) insoles wear time (≥ 80% of weeks with a minimum 210 min of wear); (iv) average comfort over the duration of the intervention (≥ 6/10); (v) diary completion (≥ 80% weekly entries completed online); and, (vi) retention (≥ 80% retained through follow‐up testing).

#### 2.8.2 Safety Outcome

The number of self‐reported adverse events that required the cessation of insoles wear for at least 2 weeks.

#### 2.8.3 Efficacy Outcomes

- Foot pain and function

The pain (7 items) and difficulty (9 items) subscales of the Foot Function Index Revised Short form (FFI‐RS) were used. The FFI‐RS exhibits excellent validity and reliability to assess foot symptoms [23, 24]. We chose to rescore all items on a 0–3 range and divide the highest possible sum by 100. This was done to achieve a true 0%–100% score, as has been suggested previously [25]. Though the activity limitations subscale (3 items) was chosen at pre‐trial registration, we later agreed that the difficulty subscale is more appropriate to comprehensively assess the effects of the insoles on the performance of activities.

ii. Knee pain and function

The pain (9 items) and function in daily living (17 items) subscales of the Knee Injury and Osteoarthritis Outcome Score (KOOS) were used. The KOOS has established validity, reliability, and responsiveness in individuals with knee OA [26, 27].

iii. Biomechanics

The main biomechanical outcome was the KAM impulse. The KAM impulse has been shown to have greater discriminative validity than the overall peak [28], and a positive association with tibiofemoral OA disease progression [7]. We also assessed the peak KAM and knee flexion moment peak and impulse to acquire a more comprehensive measure of knee loading [29], and the peak and excursion (initial contact to peak) of subtalar/rearfoot motion to assess frontal plane foot and ankle kinematics.

### 2.9 Statistical Analysis

Descriptive statistics were used to assess our feasibility outcomes with *a‐priori* criteria described above. To assess whether any changes in clinical and biomechanical outcomes align with existing literature, efficacy outcomes were assessed using two‐factor (Group and Time) general linear models with no adjustments for multiple comparisons. Estimates of within‐group effects were determined using mean changes with 95% confidence limits by comparing values at Week 12 and Baseline. All statistical analyses were conducted using SPSS v27 (IBM Corp., Armonk, NY).

## 3 Results

**FIGURE 3:**
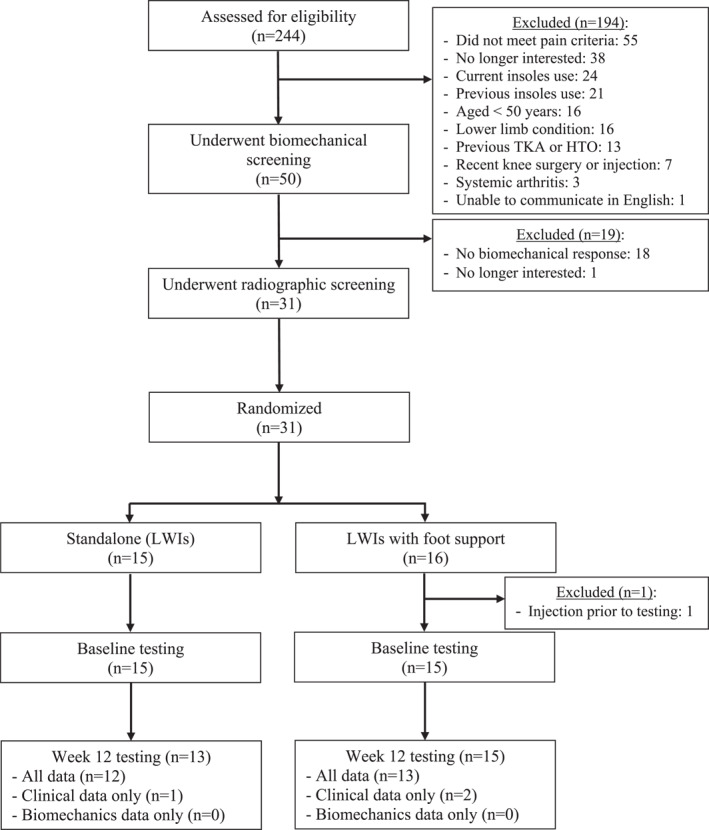
Participant flow chart.

Recruitment occurred between March 2024 and October 2025. A total of 244 individuals expressed interest, of which 31 were ultimately randomized, 30 underwent baseline testing, and 28 provided at least some Week 12 data (Figure 3). Participant characteristics are summarized in Table 1. The group with the supported LWIs contained a higher proportion of females, as well as all three participants with KL grade 4.

**TABLE 1:** Participant characteristics at baseline.

	All participants (*n* = 30)	LWI alone (*n* = 15)	LWI with support (*n* = 15)
Age (years)	64.4 (8.7)	65.0 (8.2)	63.9 (9.3)
Sex, female, *n* (%)	21 (70)	9 (60)	12 (80)
Height (m)	1.66 (0.08)	1.68 (0.10)	1.64 (0.06)
Body mass (kg)	77.7 (15.7)	78.9 (16.4)	76.5 (15.5)
KL grade, *n* (%)			
2	14 (47)	6 (40)	8 (53)
3	13 (43)	9 (60)	4 (27)
4	3 (10)	0 (0)	3 (20)

*Note:* Data are reported as mean (SD), unless otherwise noted.

All feasibility criteria were met (Table 2). Our recruitment strategy was successful in meeting our targets, with an acceptable average of 6 enrollees per recruitment drive. We were also able to meet our target delivery time for the manufacture and delivery of the insoles, with insoles delivered an average of 21 days post‐request (min, max: 10, 48 days), and all but four being delivered within the 28‐day target window. Participants exhibited excellent adherence to the intervention across multiple outcome measures. Of those who completed the study, the majority met the minimum wear times (278 of 324 weeks overall, 83%), with most of those individuals greatly exceeding the 30 min per day requirement. The insoles were perceived as moderately comfortable across all weeks (mean = 7.0/10), with higher values in Week 12 (mean = 8.0/10) than in Week 1 (mean = 5.6/10). Completion of the online diaries was also high (319 of 336 weeks, 95%).

**TABLE 2:** Feasibility outcomes.

	Acceptable threshold value	All participants	LWIs alone	LWIs with foot support
Recruitment per advertisement	5	6	n/a	n/a
Delivery time, median (IQR) days	28	21 (16, 27)	16 (13, 26)	22 (18, 27)
Wear time ≥ 210 min, *n* (%) weeks	80%	278 (83)	125 (80)	153 (85)
Insoles comfort (0–10), median (IQR)	6	7 (5, 9)	7 (6, 9)	7 (5, 9)
Online diary completion, *n* (%) weeks	80%	319 (95)	142 (91)	177 (98)
Retention to week 12, *n* (%) participants	80%	28 (93)	13 (87)	15 (100)

Most self‐reported adverse events were relatively minor, and were confined to the feet and lower legs. Ten participants (six in the supported insoles group) reported mild foot and lower leg discomfort within the first week that subsided within two to 4 weeks. Four participants reported issues lasting the duration of the intervention (two with plantar heel pain, both in the supported insoles group, one of which required cessation of insoles use between Weeks 5 and 7; two with toe numbness and tingling, both in the standalone LWIs group, neither of which required cessation of insoles use).

All but two participants (both in the standalone LWIs group) provided some Week 12 data, with most (*n* = 25) providing full datasets at Baseline and Week 12. Three participants (two in the supported insoles group) completed the KOOS and FFI at both testing sessions, but did not undergo biomechanical assessment at Week 12. Efficacy outcomes are summarized in Table 3 and Supporting Information S2: Figures S1–S5, which show changes in foot and knee function, knee pain, as well as biomechanics at Week 12.

**TABLE 3:** Within‐group differences for efficacy outcomes.

	LWIs alone			LWIs with foot support		
	Baseline	Week 12	Mean change	Baseline	Week 12	Mean change
KOOS pain (0–100)	59.6 (3.9)	74.1 (4.5)	14.5 [5.3, 23.7]	58.0 (3.4)	70.4 (4.2)	12.4 [3.8, 21.0]
KOOS daily function (0−100)	68.3 (4.2)	84.6 (4.0)	16.3 [8.2, 24.4]	63.7 (3.9)	77.6 (3.8)	13.9 [6.4, 21.5]
FFI pain	25.1 (5.2)	23.6 (7.6)	−1.5 [−15.8, 12.8]	44.0 (4.8)	30.7 (7.0)	−13.3 [−26.7, −0.1]
FFI difficulty	29.5 (7.3)	16.4 (7.4)	−13.1 [−22.5, −3.6]	44.9 (6.8)	33.8 (6.9)	−11.1 [−19.9, −2.3]
Gait speed (m/s)	1.14 (0.06)	1.16 (0.06)	0.02 [−0.01, 0.04]	1.19 (0.06)	1.19 (0.06)	0.00 [−0.03, 0.03]
Ankle/subtalar eversion						
Peak (°)	2.7 (0.8)	5.8 (1.0)	3.1 [0.7, 5.5]	4.1 (0.8)	5.7 (1.0)	1.6 [−0.7, 3.9]
Excursion (°)	5.5 (0.6)	6.7 (0.5)	1.1 [−0.2, 2.6]	6.8 (0.6)	7.3 (0.5)	0.5 [−0.8, 1.8]
KAM						
Peak (Nm/kg)	0.37 (0.03)	0.34 (0.03)	−0.03 [−0.06, 0.00]	0.42 (0.03)	0.39 (0.03)	−0.03 [−0.06, 0.00]
Impulse (Nm/kg·s)	0.14 (0.02)	0.12 (0.02)	−0.02 [−0.03, −0.00]	0.16 (0.02)	0.15 (0.02)	−0.02 [−0.03, −0.00]
KFM						
Peak (Nm/kg)	0.54 (0.07)	0.56 (0.07)	0.02 [−0.02, 0.07]	0.40 (0.07)	0.45 (0.06)	0.05 [0.01, 0.10]
Impulse (Nm/kg·s)	0.08 (0.01)	0.09 (0.01)	0.01 [−0.00, 0.02]	0.06 (0.01)	0.07 (0.01)	0.01 [0.00, 0.02]

*Note:* Session values are reported as mean (SE). Mean changes and differences are reported as mean [95% CI]. Higher KOOS scores and lower FFI scores denote better pain and function. Mean change = Week 12—Baseline. Biomechanics data are presented as the no insoles condition at Baseline and the insoles condition at Week 12.

## 4 Discussion

Lateral wedge insoles, with or without foot support, may play a supplemental role in the clinical management of knee OA. Our study methods were feasible, and participants were able to safely complete the intervention and testing. Our findings do not suggest negative foot consequences with LWIs use for those with knee OA and concurrent foot pain, and justify conducting a larger study in this subgroup.

All feasibility criteria were met, with some opportunities for improvement identified. We achieved success in recruitment despite using only one method—social media postings. In order to enhance the clinical and biomechanical diversity of the sample [30], additional recruitment approaches should be used in future trials. Participants reported that using the online diaries was intuitive and enhanced their involvement with the study, especially given the minimal interactions with study personnel after baseline testing. Participants likely would have benefited from a visit to a foot healthcare specialist within the first week of wear for more targeted fitting and wear instructions. Though very few adverse events were reported, with most limited to “discomfort” early in the intervention and lasting no more than two to 4 weeks, one common theme was a perception of “tightness” in the shoes brought on by the insertion of the insoles. Shoe fit issues are common with insoles [31], especially in those who are using them for the first time. Our attempt to minimize participant burden at study outset resulted in the decision to have the insoles sent directly to the motion analysis laboratory. Subsequent studies in this area will need to balance the time and resource burden with maximizing support and guidance in the early stages of interventions.

The overarching premise for this line of research was a need to examine the safety of LWIs use in people with knee OA and foot pain. Given the primary focus on improving knee biomechanics and symptoms, it is reasonable that outcome assessment in LWIs research has been predominantly at the knee. Unfortunately, very little is known about the effects of LWIs on the feet of people with knee OA, and nothing specifically in those who exhibit foot pain. Our findings suggest LWIs with or without supports are generally safe to use by people with knee OA and concomitant foot pain.

While neither insole type was designed to improve any foot symptoms, it is important to identify if the use of LWIs creates any new non‐knee issues in this subgroup given the known links between foot pain and outcomes relevant to knee OA [17, 19]. We were unable to statistically confirm changes in foot pain in either group, though we did see a mean decrease in pain in the supported LWI group suggestive of the potential for improvement with this insole type. Further, we observed modest improvements in self‐reported foot function in both groups. These small changes are consistent with previous research in people with knee OA and flat feet using similar insole designs [32], and supports the rationale for a trial fully‐powered on foot symptoms with the use of LWIs by people with knee OA and foot pain.

We also observed within‐group changes in biomechanical outcomes in both groups. Average KAM reductions of 10% in the impulse and 7% in peak magnitudes are slightly larger than the ranges reported in previous studies [10, 11]. This was likely the result of our pre‐screening approach that ensured that only those who exhibited a KAM reduction with LWIs were enrolled. This approach has been used previously, with beneficial symptomatic findings as well [16]. Consistent with previous research is the finding of change in ankle/subtalar peak eversion with the standalone LWIs, but no change with the supported insoles [33]. While our efficacy findings overall are representative of the known effects of LWIs for knee OA, it must be restated that the goal of this feasibility trial was to show ability to collect biomechanical and clinical outcomes in this subgroup of the knee OA population, and not test for efficacy, thus these outcomes should be interpreted with caution.

Our study is not without limitations. First, we chose to not include a flat or inert control insole condition. Given the focus on feasibility, we decided to allocate participants to only two conditions. While we cannot rule out similar effects with flat insoles, the information gained from this study will be important to inform future LWIs research with a focus on outcomes distal to the knees. Second, our biomechanical screening was conducted with the standalone LWIs only. This approach was chosen to minimize costs and time. Specifically, having a number of standalone LWIs of varying lengths on hand at the first laboratory visit meant that biomechanical screening could be done without having to manufacture and deliver supported LWIs for everyone. It also avoided the manufacture of the more expensive supported LWIs for those who were not biomechanical responders to LWIs in general, or who were not ultimately randomized to the supported LWI condition. Given the expected lower KAM reduction with supported LWIs, we decided to increase the threshold for response to 4% rather than the 2% used previously [16]. However, the assessment of biomechanical response was still reliant upon laboratory‐based motion capture systems that may not be present in all clinical settings. Further development of clinically‐accessible methods to assess biomechanical response to LWIs would enhance clinical utility.

## 5 Conclusion

Further research into LWIs use in people with knee OA and foot pain is warranted and feasible. On the whole, our participants did not experience negative effects in their feet with the insoles. Data from this pilot feasibility study can be used to inform sample size requirements and protocols to better assess the appropriateness and efficacy of LWIs use in this important subgroup of the knee OA population.

## Author Contributions

**Michael A. Hunt:** conceptualization, formal analysis, funding acquisition, methodology, project administration, resources, supervision, writing – original draft. **Natasha M. Krowchuk:** data curation, project administration, writing – review and editing. **Haocheng Zhang:** data curation, formal analysis, writing – review and editing. **Michael B. Ryan:** methodology, resources, writing – review and editing. **Trevor B. Birmingham:** funding acquisition, methodology, writing – review and editing. **Jesse M. Charlton:** funding acquisition, methodology, software, writing – review and editing.

## Conflicts of Interest

M.B.R. is employed by Kintec Footlabs Inc., a company providing orthotic services. However, he does not receive any direct benefit from this research that could potentially bias these study results. The authors declare no other professional or financial affiliations that may be potential conflicts of interest.

## Supporting information


Supporting Information S1

Supporting Information S2

## Data Availability

Data available on request from the authors.
