# Supplementary material for: Lateral Wedge Insoles With and Without Contoured Arch Support for People With Knee Osteoarthritis and Foot Pain: A Pilot, Feasibility Randomized Controlled Trial
Source: J Foot Ankle Res. 2026 Jul 13;19(3):e70171. doi: 10.1002/jfa2.70171 (PMC13365356; doi:10.1002/jfa2.70171)
Supplement: Supplementary file 1 — Supporting Information S1 [file JFA2-19-e70171-s001.pdf]

Supplemental File 1. Description of biomechanical analysis for Baseline and Week 12 data.

A scaled multilink lower body model (pelvis, thighs, shanks, feet) was used to estimate segment and joint kinematics using 6 degrees of freedom at each joint. Segment coordinate frames were defined with the x-axis directed mediolaterally to the right, the y-axis directed posteroanteriorly, and the z-axis following the right-hand rule, directed inferior-superiorly. Euler angles were calculated for each joint as the distal with respect to proximal segment (order: XYZ). Net joint moments were calculated using an inverse dynamics approach and resolved in the proximal segment local coordinate system. A virtual foot segment was defined in the ground plane of the lab such that the static calibration represented neutral ankle joint angles. The joint moment impulse was calculated using the trapezoidal method and peaks were the maximum magnitude across stance phase.
