# Supplementary material for: Lateral Wedge Insoles With and Without Contoured Arch Support for People With Knee Osteoarthritis and Foot Pain: A Pilot, Feasibility Randomized Controlled Trial
Source: J Foot Ankle Res. 2026 Jul 13;19(3):e70171. doi: 10.1002/jfa2.70171 (PMC13365356; doi:10.1002/jfa2.70171)

Supplementary Figure 1. Knee Osteoarthritis Outcome Severity Score (KOOS) boxplots. Group KOOS pain and activities of daily living function (ADL) data for the supported LWIs (purple) and standalone LWIs (green) at Baseline and Week 12. Higher scores indicate less pain and more function.

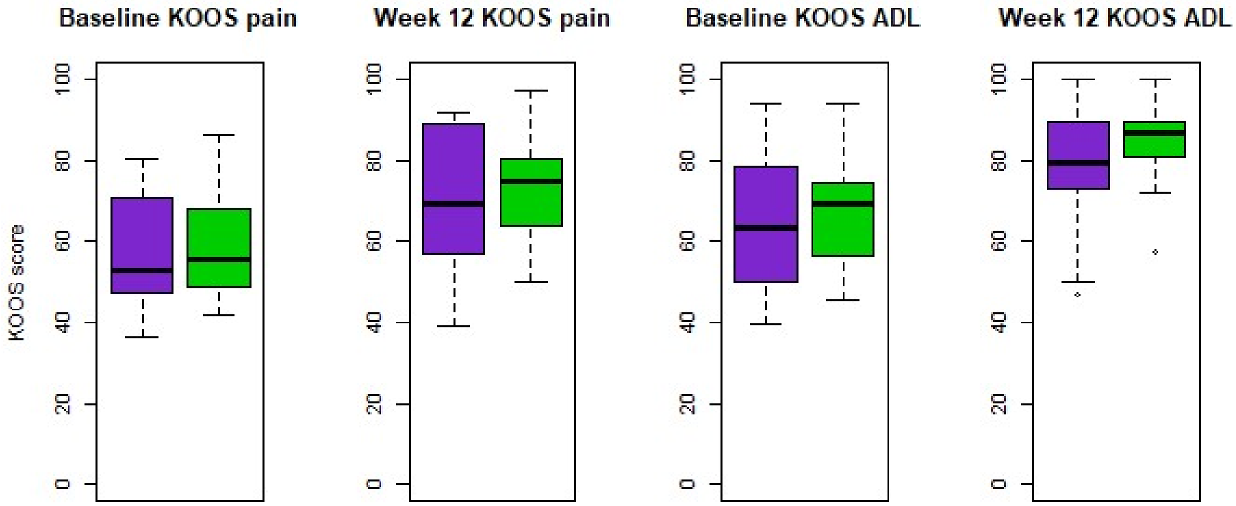

Supplementary Figure 2. Foot Function Index (FFI) boxplots. Group FFI pain and difficulty data for the supported LWIs (purple) and standalone LWIs (green) at Baseline and Week 12. Lower scores indicate less pain and more function.

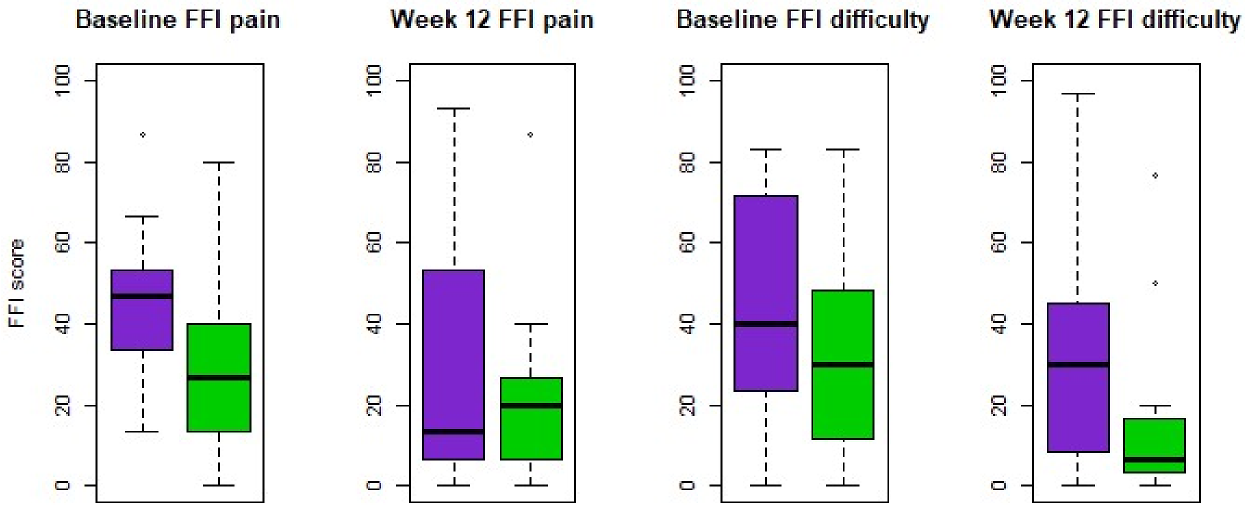

Supplementary Figure 3. Ankle eversion boxplots. Group ankle eversion peak and excursion data for the supported LWIs (purple) and standalone LWIs (green) at Baseline and Week 12.

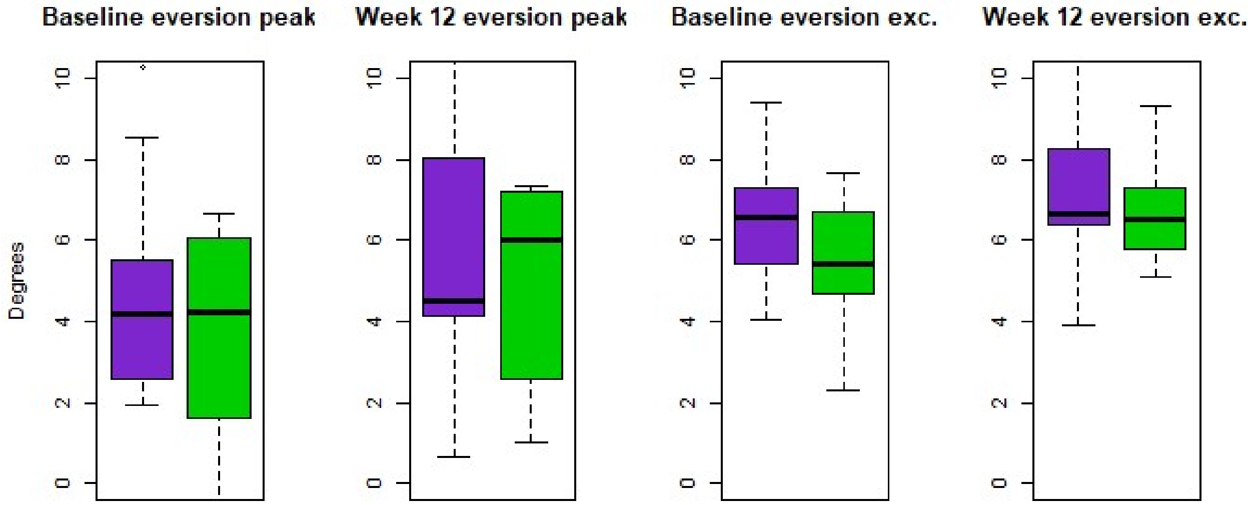

Supplementary Figure 4. Peak moment boxplots. Group peak knee adduction (KAM) and flexion (KFM) moment data for the supported LWIs (purple) and standalone LWIs (green) at Baseline and Week 12.

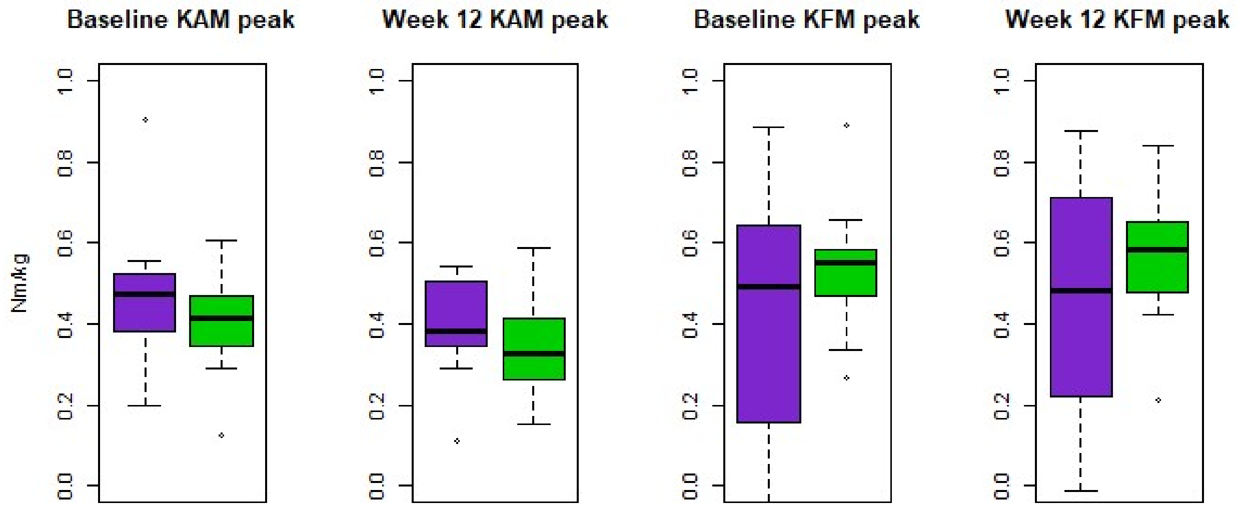

Supplementary Figure 5. Moment impulse boxplots. Group knee adduction (KAM) and flexion (KFM) moment impulse data for the supported LWIs (purple) and standalone LWIs (green) at Baseline and Week 12.

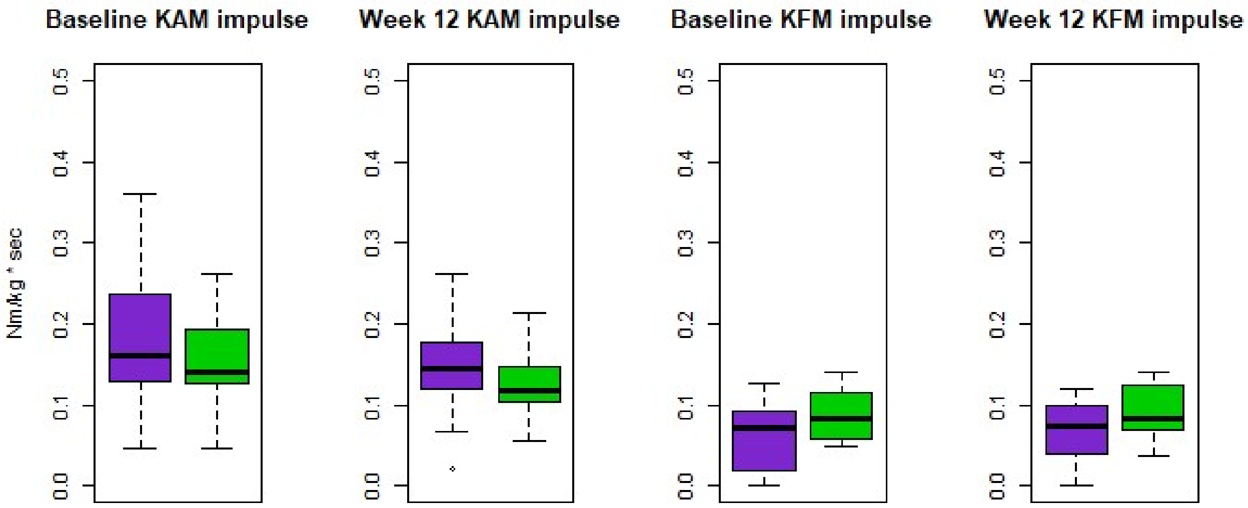

Supplement: Supplementary file 2 — Supporting Information S2 [file JFA2-19-e70171-s002.pdf]
